# A Rare Presentation of Idiopathic Unilateral Hypertrophic Olivary Degeneration

**DOI:** 10.7759/cureus.52251

**Published:** 2024-01-14

**Authors:** Lawrence T Matiski, Anish Bhandari, Hasan T Ozgur, Samuel N Rogers

**Affiliations:** 1 Diagnostic Radiology, University of Arizona College of Medicine - Tucson, Tucson, USA

**Keywords:** mr brain, inferior olivary nucleus, unilateral hypertrophic olivary degeneration, nonlesional hypertrophic olivary degeneration, idiopathic hypertrophic olivary degeneration, hypertrophic olivary degeneration

## Abstract

Hypertrophic olivary degeneration (HOD) is a rare form of trans-synaptic degeneration affecting the inferior olivary nucleus (ION). Its classical description involves a lesion in the Guillain-Mollaret triangle (GMT), characteristic imaging findings, and associated oculopalatal tremor. However, understanding of this disease entity is incomplete, as its overall rarity has limited strong classification. Case reports and small studies indicate that a variety of presentations can occur, including non-existent or non-classical lesions as well as variations in physical symptoms. Here we report the exceedingly rare case of idiopathic, nonlesional, unilateral HOD in a female patient.

## Introduction

This case report was previously presented as a digital poster at the 2023 Western Neuroradiological Society meeting on October 19, 2023. 

Hypertrophic olivary degeneration (HOD) is a rare neurological condition characterized by a unique pattern of trans-synaptic degeneration of the inferior olivary nucleus (ION) often with the involvement of a lesion in the Guillain-Mollaret triangle (GMT); however, instances of nonlesional HOD have been reported [1]. The GMT, also known as the dentato-rubro-olivary pathway (DROP), is a set of structures that play a vital role in motor coordination and control. The three corners involve the contralateral dentate nucleus (DN), ipsilateral red nucleus (RN), and ipsilateral ION [2]. The DN connects to the RN via the dentatorubral tract in the superior cerebellar peduncle (SCP) and the RN connects to the ION via the central tegmental tract (CTT) [2]. Lesions involving any of these structures may disrupt the triangle, leading to disinhibition of the ION and subsequent delayed hypertrophy due to the loss of afferent GABA-ergic neurons on the ION [3]. The hypertrophic changes in the olivary nucleus may take months to years to become apparent on MRI (magnetic resonance imaging) after the initial insult [3,4]. The olivodentary tract completes the GMT, but damage to this tract classically does not lead to HOD [5].

In this case report, we present an exceptionally rare case of unilateral idiopathic HOD with an atypical presentation and discuss the persistent uncertainties in the pathophysiology and treatment of HOD.

## Case presentation

**Figure 1:**
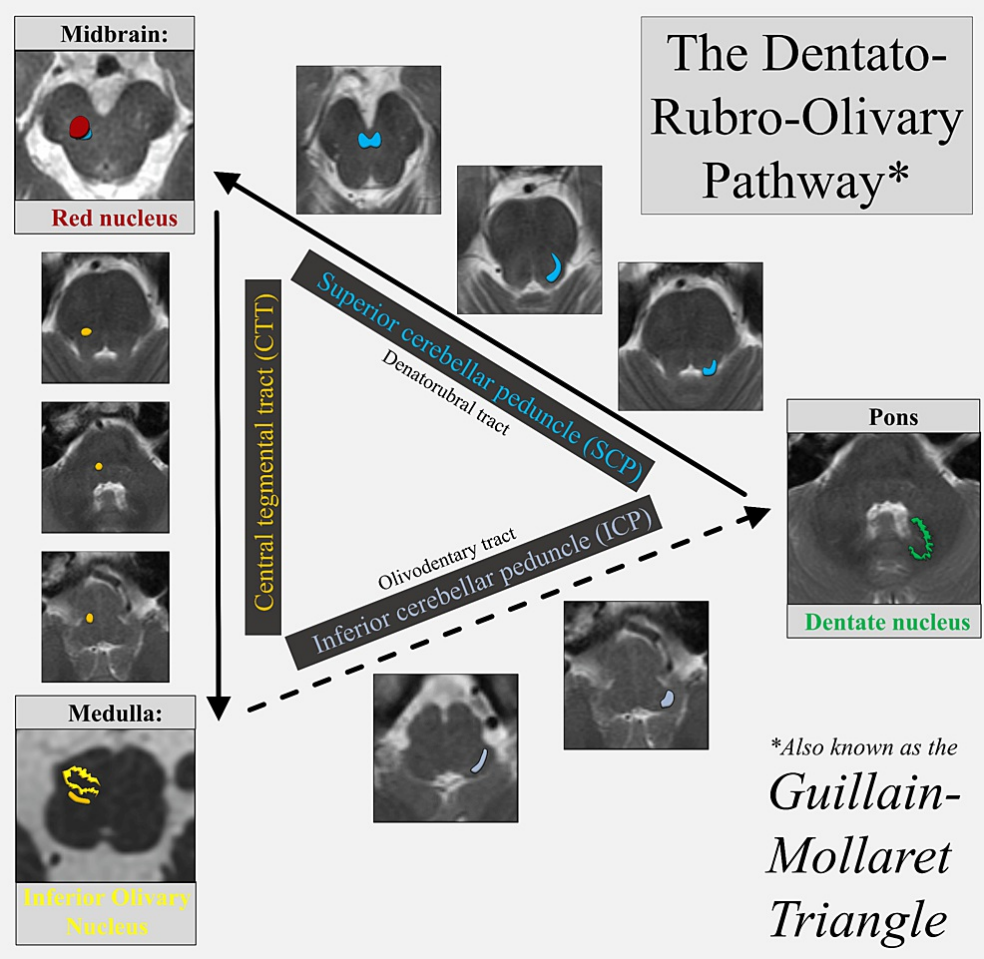
DROP Solid arrows indicate the classic locations along the DROP to cause HOD: lesions at the DN, the denatorubral tract via the SCP, the RN, and the CTT. The dashed arrow indicates efferent fibers from the ION completing the GMT, decussating via the inferior cerebellar peduncle along the olivodentary tract to the contralateral RN; this tract is dashed because lesions here do not classically cause HOD. ION, inferior olivary nucleus; GMT, Guillain-Mollaret triangle; DROP, dentato-rubro-olivary pathway; DN, dentate nucleus; RN, red nucleus; SCP, superior cerebellar peduncle; CTT, central tegmental tract; HOD, hypertrophic olivarian degeneration

**Figure 2:**
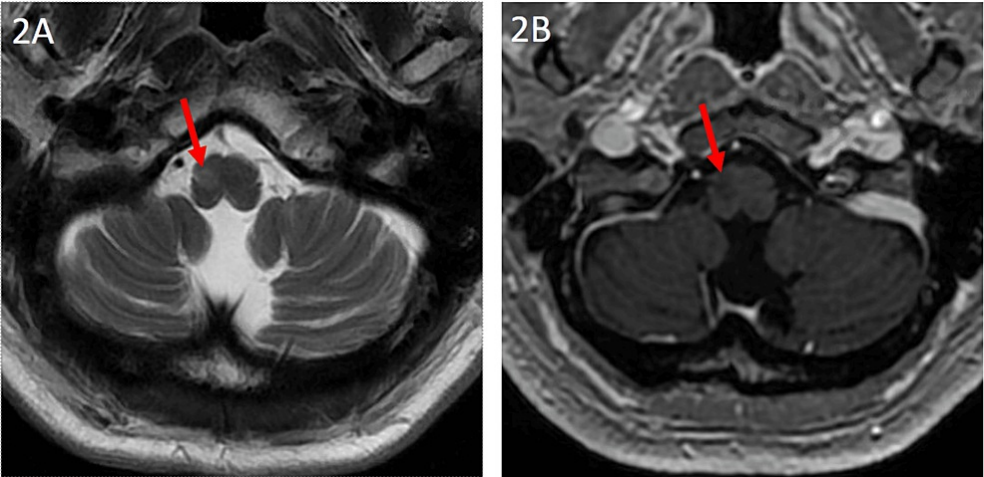
Imaging after initial symptom onset Axial T2 (2A) and post-contrast MPRAGE T1 (2B) weighted images demonstrate mild enlargement of right ION, which is slightly hyperintense on T2 weighted images and hypointense on MPRAGE T1 weighted images. There was no contrast enhancement.​ ION, inferior olivary nucleus

A 65-year-old female with a history of developmental delay, epilepsy, and invasive ductal carcinoma (IDC) currently in remission on aromatase inhibition presented with one month of progressive difficulty in balance and left-sided ataxia. Physical exam showed slightly decreased reflexes in the lower left leg and a slow unsteady gait, though cerebellar testing was intact bilaterally and no tremor or rhythmic movements were present. Given her pre-existing epilepsy and cancer diagnoses and new onset neurological findings, further workup was performed. Spot EEG without accompanying seizure showed the abnormal pattern of generalized intermittent rhythmic delta activity, a nonspecific indicator of encephalopathy [6]. However, the patient did not display altered consciousness, reported being seizure-free for four years while off anti-epileptic medications, and showed no significant abnormalities on basic laboratory workup. MRI findings revealed a non-enhancing T2 hyperintense lesion in her right medulla oblongata suggestive of HOD, though the classical GMT pathway showed no apparent lesions (Figures 1 and Figure 2).

**Figure 3:**
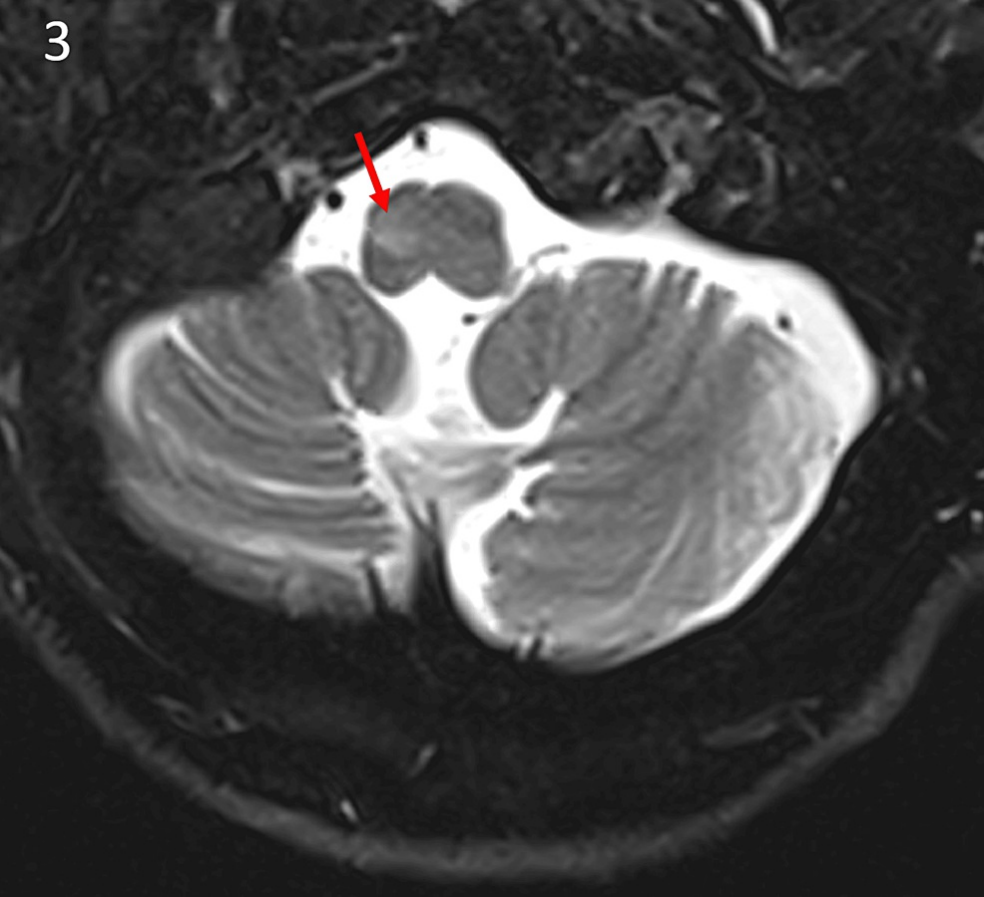
Imaging two months post-initial symptom onset Axial T2 weighted imaging two months after demonstrates no change.

Similarly, there were no other detectable lesions or apparent encephalopathic changes in the surrounding areas. Notably, no diffusion restriction, susceptibility, or post-contrast enhancement occurred in the region, making infarct, hemorrhage, infectious, or neoplastic processes less likely [7]. The patient was offered but declined anti-epileptic medications for symptom management and was offered close follow-up and a referral to neurosurgery if symptoms worsened. After two months, the physical exam and imaging were repeated, showing the stability of her physical symptoms and the non-enhancing T2 hyperintensity (Figure 3). The patient had no additional complaints by the 10-month point and oncology follow-up continued to show no concern for recurrence of IDC. The patient was continued on the aromatase inhibitor exemestane. It should be noted that she had prior treatment with the aromatase inhibitor letrozole years before the appearance of the above neurological symptoms.

## Discussion

Traditionally, unilateral HOD is secondary to a lesion in the ipsilateral CTT or contralateral DN [7]. Bilateral HOD is classically secondary to a lesion large enough to affect both the SCP and CTT, thereby disrupting bilateral DROPs [7]. The etiology of HOD is diverse and can be due to various factors, including vascular, neoplastic, traumatic, infectious, or degenerative causes, though can also remain idiopathic [8]. Several rare mitochondrial disorders have also been associated with typically bilateral HOD, though the lack of widespread systematic gene sequencing likely conceals their overall frequency [8].

HOD’s range of presentations is not well understood. A variety of clinical presentations ranging from asymptomatic to severely disabling are possible. The clinical impact of symptomatic HOD can be diverse, with symptoms including palatal myoclonus, oscillopsia, and upper extremity tremors [3,8,9]. This necessitates individualized patient management that can involve a variety of antiepileptic medications, botulinum toxin injections, resection, and deep brain stimulation [2,10]. Patients display variable responses to pharmacologic and neuro-interventional approaches [10]. Some cases appear to be self-limiting, though the process is not currently understood, and no treatment regimen has yet been identified as consistently effective or optimal for all cases of HOD [10]. 

Though HOD can be suspected due to clinical impression, a definitive diagnosis requires characteristic MRI findings. HOD imaging findings consist of T1 nonenhancement and T2 hyperintensity in one or both IONs [4]. The characteristic T2/FLAIR hyperintensity occurs early, potentially as soon as one month after a known lesion occurs in the dentatorubral tract, and remains indefinitely [4]. Hypertrophy of the ION occurs at about six to 12 months and usually transitions to atrophy after three to four years, although these timelines are variable [3-5]. HOD diagnosis also requires the T2 hyperintensity to be T1 non-enhancing, as enhancement can represent other etiologies such as inflammation or neoplasm [7]. Other MRI sequences may become more relevant to identifying HOD in the future if quantitative approaches can be developed, but a small study involving a direct comparison between the current T2 hyperintensity methodology, proton density, and DWI-based approaches showed the best results in identifying HOD via detection of T2 hyperintensity [11]. The current literature on HOD is limited and is largely confined to case reports, case studies, and research studies limited to patient numbers in the low hundreds [12]. Overall understanding of this phenomenon is limited by a general lack of data, and we thus report an incidence of the already rare HOD that current evidence indicates is even rarer still.

Data on HOD is limited to case reports and small case series [2,12]. HOD has been reported to be more common unilaterally, though more recent larger analyses of data sets from two institutions indicate it may be more common bilaterally [1,10,13,14]. These more recent analyses also challenge the traditional association of HOD with lesions in the GMT, as almost half of HOD patients may not have a lesion within the GMT, and a smaller proportion (23/116) may truly have nonlesional HOD, i.e., without lesions present anywhere in the brain [1,13]. However, what is consistent across both recent compilations of case reports and larger data set analyses is the overall rarity of unilateral, nonlesional, idiopathic HOD. A case report compilation shows only three cases of idiopathic HOD, and all cases were bilateral [10]. The larger studies all drew from the same institution’s data set, potentially limiting their results, but all found nonlesional HOD to be largely bilateral [1,13,14]. Of the rare nonlesional, unilateral HOD patients, four out of six were male [13]. Our patient’s symptoms are not a classical ocular or palatal tremor, again distancing her from the classical description of HOD. Thus, we have identified a female patient with nonlesional, unilateral HOD creates a presentation that, while not truly unique to the medical literature, has only been minimally reported in larger studies but not in individual case reports.

## Conclusions

We present a rare form of HOD: unilateral, nonlesional, idiopathic HOD in a female patient with atypical symptoms. Initial workup involved concern for a seizure-related or metastatic process, though non-specific EEG findings, no significant laboratory abnormalities, lack of lesional contrast enhancement on MRI, and continued non-concern from oncology follow-up, all in the context of MRI findings classical for HOD, indicated that the patient’s presentation was most consistent with HOD. Per the patient's request, she was managed conservatively and did not display the progression of symptoms or imaging findings.

A brief literature review reveals substantial contradictions in current reports and studies on HOD, with significant gaps present in terms of epidemiology and understanding of the disease process and optimal treatment. This report, though potentially limited by the patient's complicated history, highlights the possible heterogeneity of symptoms in a patient with an unexpected diagnosis of HOD. We further discuss some possible differential diagnoses of HOD and the uncertainty surrounding this disease process and illustrate the need for further research and systemic review. 
